# Developing and validating high-value patient digital follow-up services: a pilot study in cardiac surgery

**DOI:** 10.1186/s12913-022-08073-4

**Published:** 2022-05-21

**Authors:** A. Londral, S. Azevedo, P. Dias, C. Ramos, J. Santos, F. Martins, R. Silva, H. Semedo, C. Vital, A. Gualdino, J. Falcão, L. V. Lapão, P. Coelho, J. G. Fragata

**Affiliations:** 1 Value for Health CoLAB, Lisbon, Portugal; 2 Comprehensive Health Research Center, Nova Medical School, Nova University of Lisbon, Lisbon, Portugal; 3 CEG-IST, Instituto Superior Técnico, University of Lisbon, Lisbon, Portugal; 4 Hospital de Santa Marta, Centro Hospitalar Universitário Lisboa Central, Lisbon, Portugal; 5 NOVA-LINCS, NOVA School of Science and Technology, Nova University of Lisbon, Lisbon, Portugal; 6 NOVA CLUNL - Center of Linguistics, Nova University of Lisbon, Lisbon, Portugal; 7 UNIDEMI, NOVA School of Science and Technology, Nova University of Lisboa, Lisbon, Portugal

**Keywords:** Digital healthcare, Service design, Remote patient monitoring, Design science research, Real-world validation, Patient-reported outcomes, Cardiac surgery

## Abstract

**Background:**

The existing digital healthcare solutions demand a service development approach that assesses needs, experience, and outcomes, to develop high-value digital healthcare services. The objective of this study was to develop a digital transformation of the patients’ follow-up service after cardiac surgery, based on a remote patient monitoring service that would respond to the real context challenges.

**Methods:**

The study followed the Design Science Research methodology framework and incorporated concepts from the Lean startup method to start designing a *minimal viable product* (MVP) from the available resources. The service was implemented in a pilot study with 29 patients in 4 iterative develop-test-learn cycles, with the engagement of developers, researchers, clinical teams, and patients.

**Results:**

Patients reported outcomes daily for 30 days after surgery through Internet-of-Things (IoT) devices and a mobile app. The service’s evaluation considered experience, feasibility, and effectiveness. It generated high satisfaction and high adherence among users, fewer readmissions, with an average of 7 ± 4.5 clinical actions per patient, primarily due to abnormal systolic blood pressure or wound-related issues.

**Conclusions:**

We propose a 6-step methodology to design and validate a high-value digital health care service based on collaborative learning, real-time development, iterative testing, and value assessment.

## Introduction

Emergent digital solutions can impact healthcare positively, but it remains a challenge for service providers and developers to demonstrate the value of their digital innovations in healthcare [1, 2]. Conventional methodologies based on high investment for technology development followed by a robust clinical study for validation fail to cope with the fast pace of digital health innovations [3]. More pragmatic approaches are needed to support evidence gathering, incremental development, and accumulated knowledge base that cope with low initial resources and gradually demonstrate the value in real-world healthcare environments to support scale-up [2–4].

Design science research (DSR) develops knowledge from the design, development, and iterative evaluation of artefacts, i.e., incrementally improved solutions to real context problems [5]. DSR methodologies have been applied to innovation in digital services for healthcare and demonstrated to allow both a theoretical and experimental approach to real-world healthcare problems [6, 7].

Patient follow-up is essential in cardiovascular patients’ health pathway [8]. In cardiac surgery, complications during surgery or hospitalization may occur [9]. However, risk also extends to the postoperative period, leading to hospital readmission of 15 to 20% of patients during the first month and 30% in the first year [10–12]. Post-discharge telemonitoring can be a valuable tool to maximize surgery outcomes [13]. Despite limited studies on such programs in cardiothoracic surgery, its use is well implemented in chronic heart failure, positively impacting the quality of life and preventing hospital readmission and mortality [14, 15]. It also allows for a reduction of costs, both for the patient (hospital commutes and consultations) and the hospital (patients’ transportation, treatment of complications, and complementary diagnostic exams) [16]. When applied to post-surgery follow-up, there are not enough studies demonstrating value from these digital services, namely reducing readmissions and costs of care [12]. Alongside, studies of postoperative patient-reported outcomes measures (PROMs) are low in volume, and evidence needs to be strengthened, namely with more digital resources [17].

This project started with minimal investment, based on the collaboration of technology partners that made available a set of Digital Health Kits (DHK), composed of smartphones with internet connection and IoT devices developed for cardiac insufficiency [18]. Driven by the need to use the available resources to develop the Remote Patient Monitoring (RPM) system in such a way that could add value to the established follow-up program, we followed a participatory approach to implement a pilot study with the active involvement of all stakeholders [19]. We used the design science research methodology (DSRM) as a baseline framework to develop the digital solution, supporting the research team to iteratively respond to the real context challenges and assess its value [5, 20]. A lean startup approach was needed to help researchers to quickly start with the minimal development needed to initiate patients’ and clinicians’ experience with the new service.

This paper presents the work undertaken to implement a postoperative digital telemonitoring service for patients submitted to cardiac surgery in Hospital de Santa Marta, a central public hospital in Lisbon, Portugal. The service was developed from existing technological equipment supplied by technology partners that supported the project.

The primary objective of this study was to develop a digital transformation of the patients’ follow-up service of a cardiac surgery department, that would collect patient reported outcomes and respond to the real context challenges. The secondary objective was to evaluate the feasibility of such digital follow-up service for delivering high-value care to the patients.

## Methods

### Methodology

This study followed the framework of DSRM and incorporated concepts from the Lean startup method [21] to start designing a *minimal viable product* (MVP) from the available equipment (DHK) and resources (provided by the clinical team and the research team). DSRM oriented the researchers to develop a rigorous design-oriented framework centered on the actual context and needs of patients and healthcare professionals by continuously communicating with them. The lean startup approach guided the team to bear uncertainty by quickly starting a pilot study and developing an overall solution in short deployment cycles. The entrepreneurial mindset drove the research team to validate learning during the DSR process and follow a “Build-Measure-Learn” sequence. During the pilot study, the overall RPM service was co-designed and developed by researchers, patients, and the clinical team, relying on short cycle times and rapid iteration with small batches of patients submitted to cardiac surgery [21–23]. We started with an MVP based on the available DHK. Furthermore, we developed a digital platform artifact to support the clinical team in managing patients’ data reported from the DHK.

**Fig. 1:**
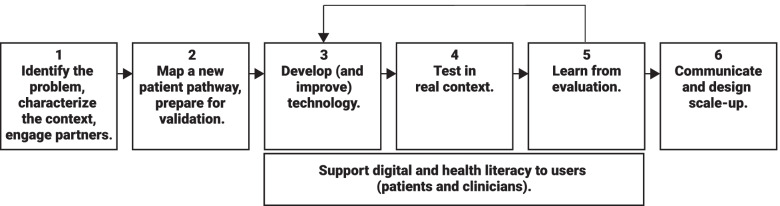
Methodology to design and develop-test-learn cycles of the RPM service for cardiac surgery follow-up

The applied methodology followed a 6-step workflow, based on the DSRM, as depicted in Fig. 1 and described below.

### Step 1 – Identify the problem, characterize the context, and engage the stakeholders

According to DSRM, the specification of the problem and motivation should anticipate any design or development to involve the teams and develop sustainable solutions. The project started with a core team of surgeons and nurses responsible for postoperative care in cardiac surgery patients. From four visits to the hospital service and conversations with the clinical team, a description of the existing postoperative follow-up procedure was made to understand the need for an RPM service that effectively collects patients’ outcomes helps to minimize post-surgery complications, and provides reassurance to patients. From each conversation, notes were taken to prepare drafts of the pathway and requirements of follow-up, that were discussed in the next meeting, until a final version was reached. Concerns on its feasibility to engage patients and nurses were discussed; patients’ adoption and nurses’ adherence to the service were set as requirements for the digital innovation. The output of this step was the design of the *as-is* patient care pathway. It describes the postoperative care program, which was agreed by the clinical team. The used process design was based on a Business Process Model and Notation (BPMN), that is used to support process modeling in healthcare and identify opportunities for quality improvement [24]. Also, we identified stakeholders relevant to the healthcare design and characterized their working needs, values, and expected benefits and contributions. This step allowed us to define a strategy for creativity of the involved stakeholders, and improvement of understanding of their potential contributions to the new digital service design. Not only were patients and clinical teams considered to participate, but technology providers who invested in this proof-of-concept were engaged and motivated towards optimal collaboration, as well.

### Step 2 – Map a new patient pathway with the clinical team for an RPM service and prepare a validation study

This step was a creative stage of the project, where, according to the DSRM, the objectives are defined. In two meetings at the hospital, we moderated a research discussion with the clinical team on use cases and scenarios for a digital remote follow-up service after cardiothoracic surgery. First, the technological opportunities (the DHK) were presented to the clinical team as the available resources to implement an RPM service. Then, the requirements for the service (target population, the outcome variables with relevance for follow-up, and the period for telemonitoring) were discussed and agreed upon by the clinical team. By suggestion of the clinical team, a patient whom we called the patient zero voluntarily tested the available technology *as-is* to assess its feasibility in terms of patient easiness-to-use and technological robustness. Finally, technology providers assessed and discussed the viability (based on the constraints of the low initial investment in technological development) of the proposed requirements. After making necessary adjustments, we discussed these with the clinical team, and a final viable consensus was reached to a new patient pathway that included a RPM service. The output of this step was a new *to-be* patient pathway and the design of a pilot study to test the new digital follow-up service. The protocol for the pilot study was submitted to the Ethical Committee of the hospital.

### Step 3–5 – Develop-test-learn in iterative cycles (DSRM cycles)

After the objectives were set and the new follow-up pathway was defined and validated by the clinical team and the technology providers, an iterative method of consecutive develop-test-learn cycles supported the development of the telemonitoring service. For each cycle, we developed new features, tested these with the participants in the study and collected their experience at the end of their 30-day follow-up period, with interviews taken in presence at the hospital.

We developed the first instantiation of the RPM service with an MVP based on minimal development efforts for a rapid move to a demonstration. The MVP was developed and tested in real context. The first patient was selected with the criteria of being admitted for cardiac surgery and having high potential motivation to collaborate on a digital remote monitoring experience. After the first MVP, we conducted significant improvements. Successive instantiations were developed, tested and evaluated with patients, following an iterative method of consecutive develop-test-learn cycles. After the 30-day telemonitoring period of each patient in each cycle, the patient experience was collected through interviews in person, at the hospital. From the reported experience, and clinicians’ feedback, we identified new requirements and defined a roadmap for further development iterations and the number of patients to test each iteration. During in presence weekly follow-up meetings with the research team, nurses and surgeons were asked for feedback on their experience with the RPM and returned necessary inputs on their needs and suggestions. These were noted and considered the priority requirements to implement in the next iteration cycle.

Software development and User Experience were relevant skills to perform the *develop-test-learn* cycles. The output of the pilot study was a telemonitoring solution with all the necessary features to make it feasible for cardiothoracic surgery care.

### Step 6 – Communicate and prepare for scale-up

According to the literature, following the DSRM, communication “establishes repeatability of the research project and builds the knowledge base for further research extensions” [5]. We developed a communication plan for each stakeholder involved to raise awareness of the project progression, opportunities, and pitfalls.

### Support digital and health literacy to patients

It has been demonstrated that patients’ active role significantly impacts the innovation of services, products, or processes in healthcare [25]. Some authors highlight responsiveness and reflectiveness to be part of the process [26, 27]. This step is fundamental to inform and involve the patient, as well [27].

A 2-page written guide illustrating the reporting procedure was delivered to the patient as supportive material. Furthermore, text messages to support health literacy during the recovery period were implemented. The nurse team defined a set of messages organized in a 30-day schedule to be sent to patients’ smartphones when they daily reported data. At the end of each telemonitoring period, when patients returned to the hospital for a clinical appointment, two researchers assessed each patient’s experience by a questionnaire and a structured interview.

### Pilot study and evaluation

#### Participants

Participants were both the clinical team responsible for the follow-up service and 30 patients. The sampling size was based on a flat rule of thumb based on theoretical optimal values of pilot trial sample size, which the clinical team considered a feasible dimension for the context of this pilot study [28].

All patients submitted to the cardiac surgery ward were eligible. The clinical team selected the patients during the post-surgery hospitalization, with the following selection criteria: ability to read and write, having a mobile phone and willingness to participate. Patients who could not manage the smartphone of the DHK, either due to functional limitations or very low digital literacy, and did not have the daily support of a caregiver, were excluded. Due to specific pandemic organization of the public health system, the hospital was COVID-19 free, i.e., patients with COVID-19 were transferred to another hospital that was referenced for that purpose.

After being selected by the clinical team, each patient was invited to participate in a 30-minute education session. One nurse and two researchers from the telemonitoring support team were present in each session. Four parts composed the session: 1) the nurse explains the project’s main goals and the telemonitoring data process and assures that the patient understands them; 2) the support team shows all the steps that the patient needs to perform when at home, and checks the patient’s ability to perform each action; 3) the patient independently repeats the routine by following the provided guidelines, and self-assessed his/her performance deciding whether he/she wants to participate, and if so, 4) the patient voluntarily signs the study informed consent, receives a 1-page instruction and the support team provides their phone contact, in case of any equipment failure or misuse. Whenever the patient was willing to participate but had very low digital literacy, the caregiver received the instructions to support the patient.

#### Instruments and evaluation metrics

The DHK included a smartphone with a SMARTBEAT app to collect data from a smartwatch (to measure steps and continuous heart rate), a sphygmomanometer (to measure blood pressure and heart rate), and a scale (to measure bodyweight) [18]. A chatbot application was also included to exchange messages and a picture of the wound. The selection of patients was independent of their home conditions related to internet connection because each kit included a 4G card to exchange data via a telecom network.

##### Patient experience

At the end of the telemonitoring program, patients’ experience was collected using a Portuguese validated version of User Experience Questionnaire (UEQ), the Net Promoter Score (NPS), and three open questions related to the recovery period: “What was most important to you during the recovery period?”, “What was most difficult?” and “What would you recommend to improve the follow-up service?” [29, 30]. NPS was used in this study as a metric for patient adoption that is simple to apply to patients with low literacy. This scale is based on a single question: “how likely are you, on a scale from zero to 10, to recommend telemonitoring to a friend or a colleague?”. Responders are grouped according to scores: promoter (9, 10), passive (7, 8) and detractor (< 7). UEQ assesses user experience and contains 26 items organized in 6 scales: attractiveness, perspicuity, efficiency, dependability, stimulation, novelty. Each item is scored from − 3 (horribly bad) to + 3 (extremely good), 0 is a neutral answer. Each scale is based on a set of items and its score is calculated as the mean of its items’ scores. The questionnaires were applied in a paper-pencil form at the hospital.

##### Feasibility of digital service

Feasibility was measured by: (i) patient’s adoption and adherence, (ii) clinical team’s adherence to the telemonitoring service and engagement (iii) the rate of technical support occurrences during the pilot. Further, we observed how the RPM service was used for clinical support to patients, by analyzing actions that were triggered by patients’ reported data.

Patient adoption was assessed with the NPS. Patient adherence was measured as the ratio of the number of days that each patient-reported outcomes to the total days that the patient had the DHK. Indicators for clinical adherence were: the number of daily accesses to the platform and the number of clinical interventions generated from data in the telemonitoring platform. Indicators for clinical engagement were: the total number of clinical users of the RPM platform.

**The clinical effectiveness** of the RPM service was analyzed by comparing the critical incidents observed in the group of patients that participated in the pilot study with a control group of patients. Each participant was compared with patients from a hospital surgery follow-up registry of critical incidents (readmissions, surgeries, death) that matched the age, sex and type of cardiac surgery.

#### Develop-test-learn cycles

During the pilot study, patients were recruited in 4 stages. Each stage corresponded to a develop-test-learn cycle, as described in previously. The defined strategy was to follow a 4-cycle iterative process, with batches of a growing number of patients: 1,5,10 and 14, respectively.

#### Data analysis

Simple descriptive statistical analysis was used for evaluating the results. We used the Wilcoxon Signed-Rank Test to compare the critical incidents of the participants with the average of the registration of critical incidents for a control group from the same cardiothoracic surgery department [31]. The control group was selected from a database of patients that were operated in the same cardiothoracic surgery department in the last 10 years. Patients in the study were compared on a 1-to-n matching, where the n was the group of patients that matched age/sex/type of surgery (the 3 main factors influencing the risk of surgery) of each participant. A *p*-value of 0.05 was considered the threshold for statistical significance.

Interviews with the patients were manually transcribed. An inductive thematic analysis was used to identify the most relevant topics raised by patients concerning the clinical support in postoperative rehabilitation.

## Results

### Implementation

This overall methodology was implemented for 16 months, from June 2019 to October 2020.

#### Step1

Stakeholders were identified and interviewed to identify their specific requirements for the context of the digital telemonitoring service. Table 1 describes each stakeholder’s expectations and their level (low/medium/high) of importance and influence in the design of the digital health service. The clinical leaders were identified as the ones with more influence in the design of the RPM service. The actual follow-up service and the process were characterized and validated by clinicians.

**Table 1:** Characterization of the stakeholders in the current project

Stakeholder group	Profile	Identification of motivations and objectives	Solution benefits	Importance	Influence	Relationship with other stakeholders
Patients	Individuals submitted to cardiac surgery and were selected for the digital telemonitoring follow-up.	Be safe and in surveillance by the clinical team; have a successful recovery.	Increased patient’s perception of safety; increased participation through recovery; better adherence to the clinical recommendations.	High	Medium	Surgeons and nurses; Family caregiver; R&D team.
Family caregiver	Patient’s relative that is providing support to the patient along with the follow-up	Support the patient in successful recovery; be supported in patient’s care. Guarantee that the patient is well treated.	Ensure the best patient recovery and clinical support; be aware of the patient’s health status; accessibility to care delivery.	Medium	Low	Patients; Surgeons and nurses; R&D team.
Surgeons and nurses from the cardiothoracic department	Healthcare professionals providing healthcare services.	Improve patients’ reassurance; improve outcomes through recovery; be informed about patient’s status to detect complications early.	Decrease the number of critical incidents; increase patient’s perception of safety; ability to close monitor a higher group of patients.	High	High	Patients; Family caregiver; R&D team.
R&D team	R&D organization that ensures that the technological pilot adds value to the digital health service provided and meets the stakeholders’ needs.	Provide a valid solution to the actual healthcare context. Study the value of a digital telemonitoring service in healthcare.	Development of new methods to effectively collect outcomes in outpatient environments; assessment of the solution’s impact.	High	High	Patients; Family caregivers; Surgeons and nurses; DHK providers.
DHK provider	Fraunhofer AICOS, an R&D organization providing technology research; Vodafone Portugal, a telecommunications operator.	Provide solutions that are usable, interoperable, safe, and compatible with medical device regulation. Provide tools for digital transformation in healthcare.	Development and validation of digital solutions; value assessment of new digital products; collaboration with partners.	High	Low	R&D team

#### Step2

**Fig. 2:**
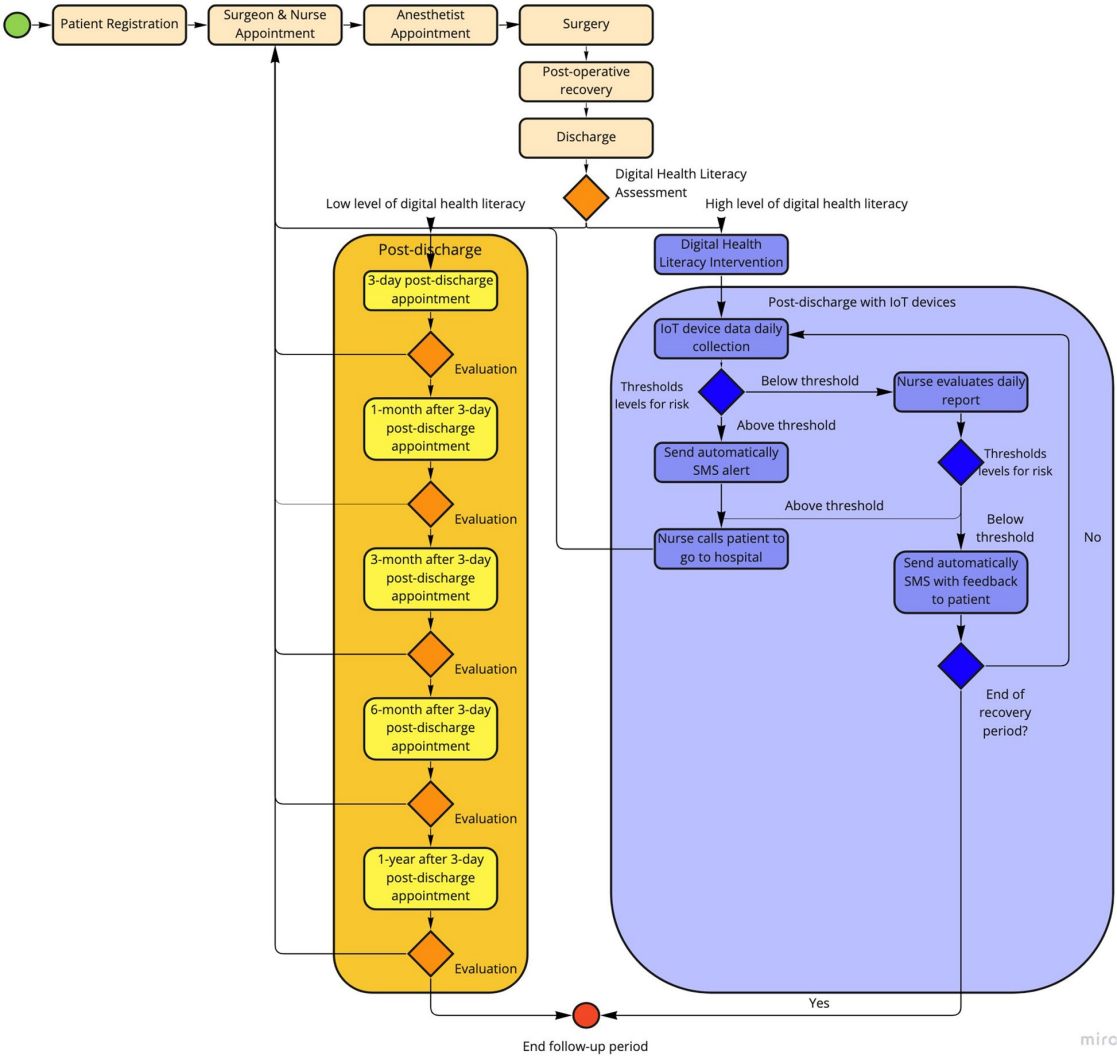
The new telemonitoring process: process underwent in the hospital (yellow) and process implemented by the RPM service (blue)

Researchers met the clinical team in three meetings to design the new pathway and define the outcomes to be monitored (Fig. 2). It was defined that patients had to report, once a day, measures that were relevant to prevent most common complications being blood pressure, heart rate, weight, and steps. Also, a picture of the wound had to be sent. Symptoms related to pain, dyspnea, feet edema, blackouts, palpitations, and wound, were collected via a 6-item questionnaire. The RPM service was set for the first month after hospital discharge, a critical period of higher complication rate. Alarm rules based on the collected outcomes were defined. The nursing team had to access to patients’ data every day, between 2 pm and 4 pm, and patients were instructed to daily report until 12 pm. Researchers worked with DHK suppliers to adjust it to the defined pathway. Moreover, the requirements and resources needed to cover the missing parts, i.e., features not implemented in the available DHK, were identified.

#### Steps 3–5

The pilot study started 3 months after the first step. Between October 2019 and October 2020, 30 patients accepted to participate in the study and used the RPM service after being discharged from cardiac surgery ward. From this group, one patient left the telemonitoring program after the first week, due to mental health conditions. The patients’ average (±standard deviation) age was 60.6 ± 13.5 years old, 15 (50%) of the participants were female. In terms of geographical distribution, half of the patients lived more than 100 km away from the hospital, the furthest district being 277 km. Patients’ average education level was 2, according to the European Qualifications Framework (EQF) (https://europa.eu/europass/en/description-eight-eqf-levels).

As we were informed, none of the patients was infected with the new coronavirus since the cardiac surgery service was set to accept non-covid patients exclusively. Patients’ recruitment was interrupted from March to May 2020 due to workflow reorganization to cope with the pandemic. Nevertheless, none of the patients already using the telemonitoring system interrupted the follow-up during that period. Besides the interruption of recruitment for 2 months, there were no other specific problems related to the COVID-19 context with the pilot study.

The DHK suffered no modifications; all patients had the same setting. This helped us to identify the need for development of an application to manage the telemonitoring data, engage the clinical team and assess value indicators. The pilot study started without developments from the application to manage patients’ data (secure links to patients’ reports were manually sent to one contact of the clinical team by email). Nurses and surgeons were proactive in suggesting features and modifications to the system or reporting satisfaction.

The iteration process, developments, and feedback used to iterate the next cycle are summarized in Table 2. Most of the developments were for the clinical side of the RPM system. The patient side of the RPM was kept the same: a smartphone to collect weight, steps, blood pressure, 6 questions related to symptoms, and the receipt of the picture of the wound. A minor change was implemented for the patient: to receive literacy text messages from nurses, just after the daily data reporting procedure. This feature was introduced in the fourth develop-test-learn cycle.

**Table 2:** Description of the four iterations performed during the pilot study to develop the digital platform for managing data from the clinical side, based on clinicians’ and patients’ feedback

Iteration	Description of the instantiation concerning the development of the data management tool for the clinical team
Iteration 1 Patient 1	Clinical team daily receives patient’s data in a report by email. Feedback from clinicians: Graphics received are not well perceived, daily reports are very extensive, historic data should be strict to a 7-day period.
Iteration 2 Patients 2–6 (1 dropout)	Clinical team daily receives patient’s data in a report by email. Improvements: Data reports were improved with the feedback given by the clinical team. Alerts were introduced in the patients’ report, based on rules defined by the clinical team. Feedback from clinicians: Need for registering clinical notes related to each patient’s reported data. Difficulty in managing information of one email (daily report) per patient. Feedback from patients: Problems with the bluetooth connection with the smartwatch was reported by some patients.
Iteration 3 Patients 7–16	Improvements: The first version of a RPM web application to manage patients’ data was launched. Data monitoring process became more efficient for the clinical team as they had a monitoring list with the individual patient alerts. Email reports were eliminated. Access to each patient record allows the access to the historic data and registering notes from the clinical team. Patient instructions were improved to reduce problems with Bluetooth connection. Feedback from clinicians: Concern of using their time in phone calls related to technical issues of the equipment. Also, text messages could save time for communicating simple literacy reminders.
Iteration 4 Patients 17–30	Improvements: Added a feature in the RPM application for sending literacy text messages to the patients. This feature included a set of predefined messages that can be sent on demand or in a scheduled scheme. Patients received the text messages once a day, after reporting the photo of the wound. To better manage the technical issues, a ticket system was added to allow the clinical team to report to our support team a problem with a DHK, avoiding phone calls. Service quantitative metrics were made available to the clinical team, in the RPM application (e.g. number of alerts and their type, type of actions taken by the clinical team based on those alerts). Feedback from clinicians: Preference for the predefined scheduled messages as can optimize their work. Feedback from patients: the app asks to daily answer to the same questions even if the answer is the same as in the previous days (“I was reporting every day that I didn’t feel tired”).

#### Step 6

Further research funding was obtained from dissemination activities to develop intelligent interaction and risk prediction from telemonitoring data. Also, communication of the results to technology partners is moving investment interest to scale up the solution.

### Support to patients’ literacy

#### Education session for digital literacy

All selected patients received the DHK and learned how to use it during the education session. During this session, one withdrawal was registered from a patient that lost interest in participating after trying the measurement procedure. Three other patients with very low digital literacy but willing to participate involved the informal caregivers in the education session to ensure data reporting with the DHK.

#### Text messages for health literacy

As a feature that was suggested by the clinical team, a list of text messages was created to send to the patients via the chatbot. These messages were based on the contents of the hospital flyer that nurses provide to the patients. Messages were divided into six categories: informative, educational/preventive, motivational, commemorative, technical support, and alert. Withal, the nurse team defined a scheduled plan to deliver recommendations for better recovery during the 30 days, weekly.

**Fig. 3:**
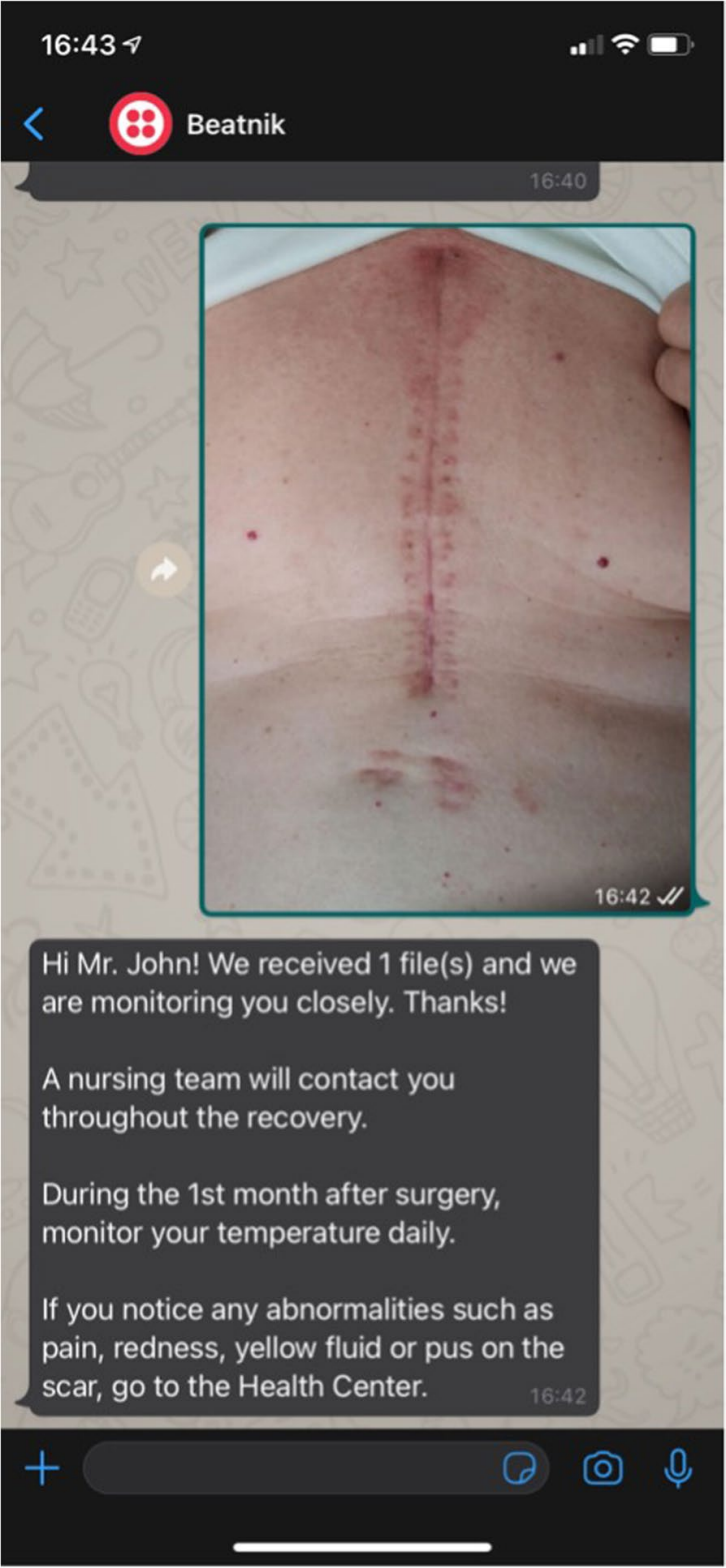
Simulation of a literacy message sent in reply to the daily picture of the surgical wound that is sent by the patients (this image includes a fiction name and is translated to English language)

Messages were implemented in the last develop-test-learn cycle, applied to 14 patients (Table 2). We divided the scheduled messages by the days of the week so patients would not receive them all on the same day. The trigger mechanism for sending the text messages was the daily receipt of the photograph of the wound from the patient, as depicted in Fig. 3. This was a deliberate strategy to increase patients’ attention to these educational messages, as these were immediately sent in response to patient’s message.

#### Evaluation

##### Patient experience

We interviewed 26 participants after the 30-day telemonitoring period. It was not possible to interview 3 of the participants on the 30-day appointment at the hospital. This is justified by difficulties in coordinating the patients’, clinical, and researchers’ schedules. Nevertheless, these participants engaged in the whole telemonitoring process and reported data that supports this study.

From the responses to the three open questions, it was observed that ten patients (38%) explicitly reported that the remote monitoring allowed them to feel safer, with 3 (12%) referring to the relevance of the collected health data and 9 (35%) highlighting the support and interest demonstrated by the healthcare professionals in their recovery. Four patients (15%) reported difficulties in using the equipment related to taking the wound pictures. It is to note that the questions did not relate directly to the DHK but to what was most relevant in postoperative rehabilitation. Some statements of the patients that relate to the use of DHK are:

- “I felt like I was at the hospital, that I was being accompanied by them all...”
- “Yesterday, I was thinking: when I get there to return the devices, I am going to have a surprise, they will give me one of this [referring to the DHK]...”
- “It is a friend that we do not see, but that is there with us every day [referring to the literacy messages in the chatbot].”

An NPS of 84 was obtained, reflecting an excellent level of patient satisfaction [32, 33]. Furthermore, 88% of patients were promoters (score of 9 or 10), enthusiastically using the technology and referring it to others, 8% were passives (score of 7 or 8), i.e., satisfied but not enthusiasts of the service, and 4% detractors (scores of 0 to 6), i.e., non-supporters of the service.

After applying the full UEQ questionnaire to 4 patients, we observed that this group of patients was not able to answer most of the questions, due to their low literacy level and complexity of the UEQ concepts. For the following participants, we applied only the scale UEQ-Stimulation (items 5,6,7,18 of UEQ), which refers to concepts that we had observed that patients could understand and relate to their experience (Table 3). From the 26 answers collected for the UEQ-Stimulation, the estimated mean value was 2.49 (confidence interval: 2.32–2.6, *p* = 0.05), as described in Table 3. The technology achieved an excellent evaluation (score higher than 1.55).

**Table 3:** Results of the Stimulation items of the UEQ

Item	Mean	Variance	Std. Dev.	Left	Right
5	2,9	0,1	0,3	Valuable	Inferior
6	1,5	1,5	1,2	Boring	Exciting
7	2,8	0,2	0,4	Not interesting	Interesting
18	2,8	0,2	0,4	Motivating	Demotivating

##### Feasibility

The mean score of NPS of 84 indicates high patient adoption of the digital telemonitoring service. Two patients even mentioned their willingness to use the DHK for some more time. Patients’ adherence was, on average, 91.6 (±15.1), all above 88 except for 4 patients: two had difficulties in using technology and were depended on caregivers’ availability, and two others had been readmitted to the hospital due to clinical complications.

Every day during the pilot study, the clinical team assessed patients’ reported data. Three nurses and two surgeons were engaged to develop the first iterations of the telemonitoring system. A scale-up was observed in iteration 4. A larger group of 25 nurses requested to be assigned to have login access to full use the web application that supported the telemonitoring process to follow patients.

Evaluation related to quantitative service metrics was only possible from cycle 3 since we had not implemented the telemonitoring management platform in the first two iterations. For the total actions registered by the clinical team on the platform, we observed an average of 7 (±4.45) occurrences per patient, most (84.3%) related to clinical interventions (Table 4). The most frequent interventions were related to nursing calls to the patient for clarification about the outcomes (measured or reported), and the least frequent were related to hospital readmission (Table 5). These interventions were triggered by data reported by the patients in the RPM system and accessed by the clinical team through the telemonitoring platform.

**Table 4:** Results of the analysis of the occurrences registered by the nurse team in the telemonitoring platform during the pilot study

Total number of occurrences registered by nurses	210
Number of clinical occurrences	177
Number of occurrences for technical support	33
Number of occurrences per patient (mean ± standard deviation)	mean (std): 7.2 ± 4.45 median (min,max): 7.0 (0,17)

**Table 5:** Description of the clinical interventions that were triggered by the telemonitoring system

Total number of interventions from the surgeon	45 (21,4%)
Medication adjustments	25 (11,9%)
Clarifications on outcomes (measured or reported)	101 (48%)
Reinforcement of education (e.g., wound hygiene instructions)	58 (27,6%)
Anticipation of the first postoperative medical consultation	4 (1,9%)
Request for reassessment of measured results	9 (4,3%)
Hospital readmission	3

Occurrences related to technical support (15,7%) were due to the synchronization of the smartwatch, or when the patient did not send the daily picture of the wound. Most of these problems were solved by phone calls between the patient and the technical support team.

Clinical interventions were labelled by nurses when they registered them in the telemonitoring application. Most of the clinical interventions were related to abnormal systolic blood pressure (19.5%) or issues related to the wound (12.9%).

The number of entries in the registry of critical incidents that matched each participant varied from 3 to 47 (mean:16,97). We used the Wilcoxon Signed-Rank Test [31] to compare the critical incidents between each participant and the mean of his/her control group. The average of critical incidents of the telemonitored group was 0.07 ± 0.26, and the average of the control group was 0.13 ± 0.13. The result of the test was w = 41 (*p*-value = 0.01), rejecting the null hypothesis that there is no difference between means.

## Discussion

This paper presents a collaborative and iterative method developed to drive a digital innovation context in a cardiac surgery department of a public hospital in Portugal. The aim was to design, implement and validate a RPM service for post-surgery follow-up.

Due to the COVID-19 outbreak, the pilot study was interrupted for just 2 months due to necessary organizational rearrangement. However, it continued with recognized added value to the patients, when there were clinical guidelines to strictly avoid hospital visits after cardiac surgery. The average age of the participants is slightly below the expected average in cardiac surgery, probably biased by the small sample and a higher willingness of younger patients to participate in the study and use the DHK. Regarding the sex distribution, it is coherent with the population in the hospital department is balanced with similar proportions of males and females [34].

DSRM enabled us to design an artifact in a participatory way, responding to the identified problem and engaging stakeholders. After the artifact was built, the lean-startup approach was used to start experimentation with a *minimal valuable instantiation*, based on available resources and minimal investment.

Our approach responds to the need to research novel methodologies to support faster technology development-validation cycles while ensuring its value demonstration. The high pace of technological innovation, in contrast to the long process of clinical validation is challenging in healthcare [1, 2]. The traditional product development process dedicates a long effort to analyzing the needs, defining the requirements, developing a prototype, and ultimately, testing and validating before market entry [35]. In healthcare, this process is heavier due to the need to validate and certificate products before reaching the market. This classical approach for product development to be validated is slow, and the delay risks digital technologies: being too late when getting to the market, being unable to prove value after a long development process, and failing to test stakeholders’ adoption in real-world settings [36, 37]. In this work, we developed the RPM service while simultaneously performing validation in different domains during the pilot study. According to the global digital health scorecard proposed by Mathews et al. [38], our methodology included the necessary elements for usability (satisfaction and adoption from patients and clinical team), clinical (reduction of critical incidents), and technical (overall performance of the system in real-world context) validation. Although it is out of the scope of this paper, we already have preliminary results of a cost analysis, which will fill the cost element of the validation scorecard [37].

The results from this study suggest the potential of patient-reported outcomes monitoring to reduce critical clinical incidents. However, a larger study is needed to support the statistical robustness of the comparison between groups of patients. Moreover, we observed that the most frequent complications solved with the RPM service in this study are comparable with a previous study, which used a similar RPM setup in post-cardiac surgery care [12]. For validation of patient experience, we found that UEQ was difficult to apply due to complex concepts that were not in the context of the patients with low literacy. Further research is needed to identify adequate instruments to evaluate the experience with technology that fits the healthcare context, being the short version UEQ-S [39, 40]. Nevertheless, the reduced rate of dropouts (only one patient) and high patient satisfaction in our study suggest that most patients’ needs were addressed.

While the clinical benefits of digital technologies in RPM have been recognized, the adoption is challenging [41]. Participatory methodologies may be a catalyzer for successful implementation and adoption of digital technologies in healthcare [42–45]. In opposition, disruption in clinical workflows by imposing an external digital tool hamper the adoption and demonstration of its value to the patients and the hospital. Accordingly, the co-design of a new digital service, along with its iterative integration in the clinical workflows, as we used, is an essential part of our contribution to optimize the feasibility and adoption of the technology [46]. Our method considered the stakeholders’ needs and motivations, the process, and the digital tools, as parts of the RPM service design. This shift from focusing on product implementation to service design has been suggested as a needed approach to ensure value co-creation through patients’ and healthcare professionals’ experiences [47, 48].

The continuous involvement of the research team with patients and clinical professionals improved their compliance with the technology, as previously identified in other studies and stressed in the Responsible Research and Innovation guidelines [1, 42, 49, 50].

The role of the researchers was also to challenge the clinical team by proposing new features and assessing acceptance and adoption. A combined strategy of demand-pull and technology-push successfully engaged the clinical team in the innovation process [48, 51]. Our iterative methodology and interdisciplinary research team stimulated innovation and promoted the adoption under this scenario.

A limitation of this study is that, despite the concern of providing a stable instantiation to the patient, the iterative method imposed different experiences to the clinical team and may hinder the reliability of the results. Another limitation is the small number of participants, which did not allow a robust statistical analysis to claim clinical effectiveness of the RPM service. Also, the small number facilitated close support of the technical team and clinicians to patients, probably increasing their adoption and masking potential outliers in terms of negative experiences with the RPM. A clinical study with higher number of patients and a stable version of the RPM service, implemented as a final iteration of our methodology, would increase the robustness of the results. From the results presented in this paper, we plan the scale-up to a randomized clinical trial with 300 patients to test the impact of a more extended digital follow-up period and analyze for which groups of patients the RPM returns higher value. Furthermore, we are working on intelligent prediction models based on patients’ outcomes, that can support a personalized care plan and high-value decisions for this follow-up service [52].

The future application of the method proposed in this work to other use-cases will be essential to validate, generalize, and improve it.

## Conclusion

We present the method we undertook to develop, implement, and validate a digital telemonitoring follow-up service in cardiac surgery care. We parted from a need to improve follow-up in cardiac surgery and the opportunity to use resources from our technological partners.

This study contributes to identifying methods that can be applied by digital health innovators, considering the demand for addressing clinicians’ and patients’ needs, available resources, and demonstration of value in short cycles of development and real-context validation.

## Data Availability

The datasets used and/or analysed during the current study are available from the corresponding author on reasonable request.

## Abbreviations

DSRM: Design science research methodology
DHK: Digital Health Kits
IoT: Internet-of-Things
MVP: Minimal viable product
NPS: Net Promoter Score
PROMs: Patient-reported outcomes measures
RPM: Remote Patient Monitoring
UEQ: User Experience Questionnaire
